# Research on the potential mechanism of Chuanxiong Rhizoma on treating Diabetic Nephropathy based on network pharmacology

**DOI:** 10.7150/ijms.47555

**Published:** 2020-08-21

**Authors:** Shanshan Hu, Siteng Chen, Zhilei Li, Yuhang Wang, Yong Wang

**Affiliations:** 1 Department of Pharmacy, Zhujiang Hospital, Southern Medical University, Guangzhou 510282, China.; 2 Department of Urology, Shanghai General Hospital, Shanghai Jiao Tong University School of Medicine, Shanghai 200080, China.; 3 Laboratory of Research of New Chinese Medicine, Zhujiang Hospital, Southern Medical University, Guangzhou 510282, China.

**Keywords:** Diabetic Nephropathy, Chuanxiong Rhizoma, Network Pharmacology

## Abstract

**Background:** Chuanxiong Rhizoma is one of the traditional Chinese medicines which have been used for years in the treatment of diabetic nephropathy (DN). However, the mechanism of Chuanxiong Rhizoma in DN has not yet been fully understood.

**Methods:** We performed network pharmacology to construct target proteins interaction network of Chuanxiong Rhizoma. Active ingredients were acquired from the Traditional Chinese Medicine Systems Pharmacology Database and Analysis Platform. DRUGBANK database was used to predict target proteins of Chuanxiong Rhizoma. Gene ontology (GO) biological process analyses and Kyoto Encyclopedia of Genes and Genomes (KEGG) pathway enrichment analyses were also performed for functional prediction of the target proteins. Molecular docking was applied for evaluating the drug interactions between hub targets and active ingredients.

**Results:** Twenty-eight target genes fished by 6 active ingredients of Chuanxiong Rhizoma were obtained in the study. The top 10 significant GO analyses and 6 KEGG pathways were enriched for genomic analysis. We also acquired 1366 differentially expressed genes associated with DN from GSE30528 dataset, including five target genes: KCNH2, NCOA1, KDR, NR3C2 and ADRB2. Molecular docking analysis successfully combined KCNH2, NCOA1, KDR and ADRB2 to Myricanone with docking scores from 4.61 to 6.28. NR3C2 also displayed good docking scores with Wallichilide and Sitosterol (8.13 and 8.34, respectively), revealing good binding forces to active compounds of Chuanxiong Rhizoma.

**Conclusions:** Chuanxiong Rhizoma might take part in the treatment of DN through pathways associated with steroid hormone, estrogen, thyroid hormone and IL-17. KCNH2, NCOA1, KDR, ADRB2 and NR3C2 were proved to be the hub targets, which were closely related to corresponding active ingredients of Chuanxiong Rhizoma.

## Introduction

Diabetic nephropathy (DN) is a common microvascular complication of diabetes mellitus (DM), which is also the most chronic complication of DM with high risk of disability and difficulty in curing 1. About 20% ~ 40% of diabetic patients are accompanied with DN 2 and suffer from increased risk of developing into end-stage renal disease (ESRD) 3. Although many therapies have been tested in animal models, there is still a lack of effective therapeutic drugs for DN since the pathogenesis of DN is very complex, which has not yet been fully understood.

Traditional Chinese medicine (TCM) has been used in the treatment of DN for years 4. Chuanxiong Rhizoma is one of these TCMs, which was originally used in the treatment of brain and heart diseases 5. Isolated from the alkaloid of Chuanxiong Rhizoma, tetramethylpyrazine has multiple pharmacological effects including anti-oxidation, improving microcirculation, and inhibiting the production of glycation 6. Many studies have revealed that Chuanxiong Rhizoma could inhibit the damage of endothelial cell 7 and the proliferation of vascular smooth muscle cell 8. It could also reduce IL-6, IL-8 and TNF-α level in serum to reduce inflammation 9. Chuanxiong injection has also been proved to provide protective effects on patients with DN through improving renal function and reducing urine protein level 10, 11. However, the mechanism of Chuanxiong Rhizoma in DN has not yet been fully studied.

Currently, network-target and multiple-component-therapeutics have been increasingly recommended in the study of TCM 12. Based on high throughput data and highly-developed algorithm, computational efforts have revolutionized the study of TCM in the ingredient identification and the target prediction. Machine learning approach has also been developed to explore the potential new interpretation of Meridians in TCM 13.

Using complex network and visualization technology, network pharmacology can display multiple targets, multiple pathways and multi-target synergy of TCM in the treatment of many diseases, which could also provide new ideas and effective measures for TCM mechanism research. In addition, multi-database exploration could facilitate mechanism understanding and drug repurposing 14. In this study, we performed a network pharmacology research focus on Chuanxiong Rhizoma to explore the possible mechanism of its functions on DN, and provide a solid theoretical basis for further study.

## Methods and Materials

### Active ingredients acquiring and target protein predicting

**Figure 1:**
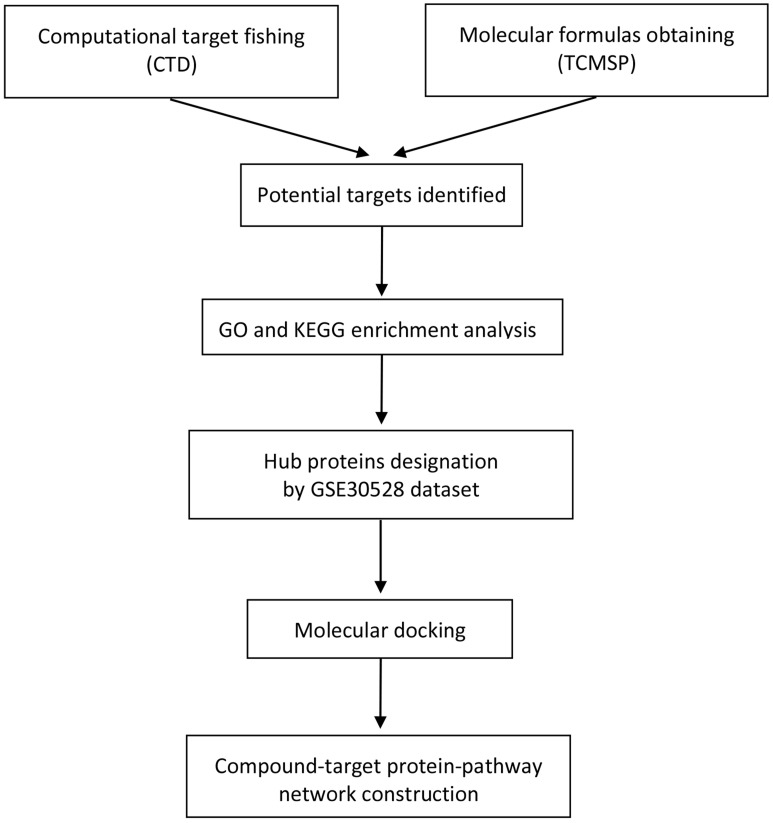
Flow diagram of the research.

Based on the Traditional Chinese Medicine Systems Pharmacology Database and Analysis Platform (TCMSP) 15 and DRUGBANK database 16, we acquired active ingredients of Chuanxiong Rhizoma by according to the strict drug screening criteria: oral bioavailability (OB) ≥ 30% and drug-likeness (DL) ≥ 0.18. The DRUGBANK database provides accurate and reliable prediction of the target proteins corresponding to chemical small molecules of active ingredients. Gene symbols of corresponding target proteins could be acquired from the uniprot database 17. The action targets associated with DN were also screened from the comparative toxicogenomics database (CTD) 18. Common target genes of Chuanxiong Rhizoma and DN were then obtained using Venn diagram. The flow diagram of this study was shown in **Figure 1.**

### Construction of target proteins interaction network

The STRING database was used to analyze the target proteins interactions. The minimum required interaction score was set at 0.7 for network diagram. The interaction network of target proteins was constructed using Cytoscape 3.5.1 software 19.

### Functional prediction of target proteins of Chuanxiong Rhizoma

Based on the interactions of target proteins and genes, we performed Gene ontology (GO) biological process analyses and Kyoto Encyclopedia of Genes and Genomes (KEGG) pathway enrichment analyses to predict the potential function of target proteins. The clusterProfiler package in R was used for statistical analysis and visualization of functional profiles for genes and gene clusters 20.

### Hub proteins designation for DN treated with Chuanxiong Rhizoma

Raw CEL data and corresponding clinical data of microarray profile from GSE30528 dataset 21 using the Human Genome U133A 2.0 Array were acquired from Gene Expression Omnibus database, and then background adjusted by Robust Multichip Average 22. GSE30528 dataset includes microarray data of 9 DN glomeruli tissues and 13 controlled normal glomeruli tissues. The limma package 23 in R environment was performed to find out differentially expressed genes (DEGs) between DN glomeruli tissues and controlled normal glomeruli tissues. DEGs with fold change of gene signature more than 1.5 or less than -1.5 were selected as potential hub genes and proteins for subsequent docking analysis.

### Molecular docking of hub proteins

In order to evaluate interactions between drug and potential target proteins, the selected hub proteins were evaluated by SystemsDock with the high-precision docking simulation assessing protein-ligand interaction 24. The molecular formulas of active ingredients were obtained from the TCMSP database. Structure files of target proteins were acquired from RCSB Protein Data Bank (PDB database) 18. Docking score, ranging from 0 to 10 representing weak combining ability to strong combining ability, represents a negative logarithm of experimental dissociation/inhibition constant value (pKd/pKi) 25.

### Statistical analysis

R 3.6.1 (www.rproject.org) and SPSS 13.0 (SPSS Inc., Chicago, IL, USA) were used for data analysis. *P* value less than 0.05 was considered significant. We also applied Cytoscape 3.5.1 software to visualize the compound-target-pathway interaction network of Chuanxiong Rhizoma in this study.

## Results

### Active ingredients and target proteins of Chuanxiong Rhizoma

**Figure 2:**
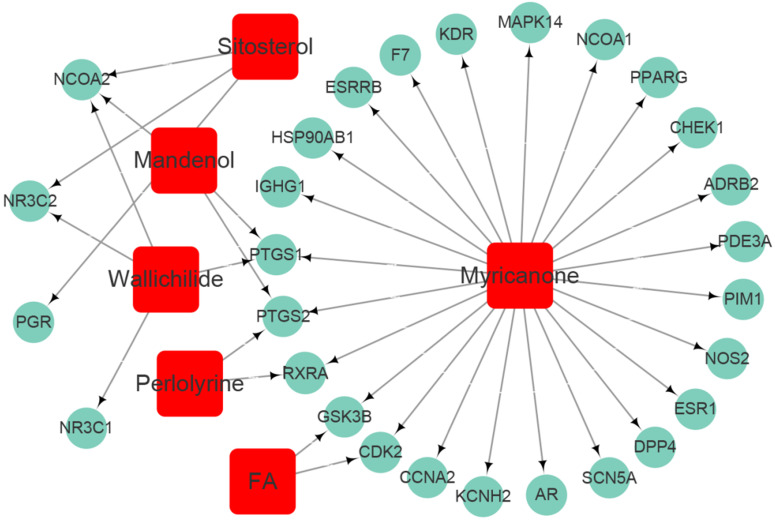
Compound-target network of Chuanxiong Rhizoma. Squires represent active compounds of Chuanxiong Rhizoma. Rotundities represent gene symbols of targets.

Seven active ingredients of Chuanxiong Rhizoma (Mandenol, Myricanone, Perlolyrine, Senkyunone, Wallichilide, Sitosterol and FA) were acquired from TCMSP database according to the suggested screening criteria: OB ≥ 30% and DL ≥ 0.18 (**Table 1**). One active ingredient (Senkyunone) without any corresponding target was excluded. Based on the reliable prediction of the target protein from the DRUGBANK database, we finally obtained 28 target proteins of Chuanxiong Rhizoma after excluding the repeated targets (**Table 2**). Compound-target network of Chuanxiong Rhizoma was shown in **Figure 2.**

**Figure 3:**
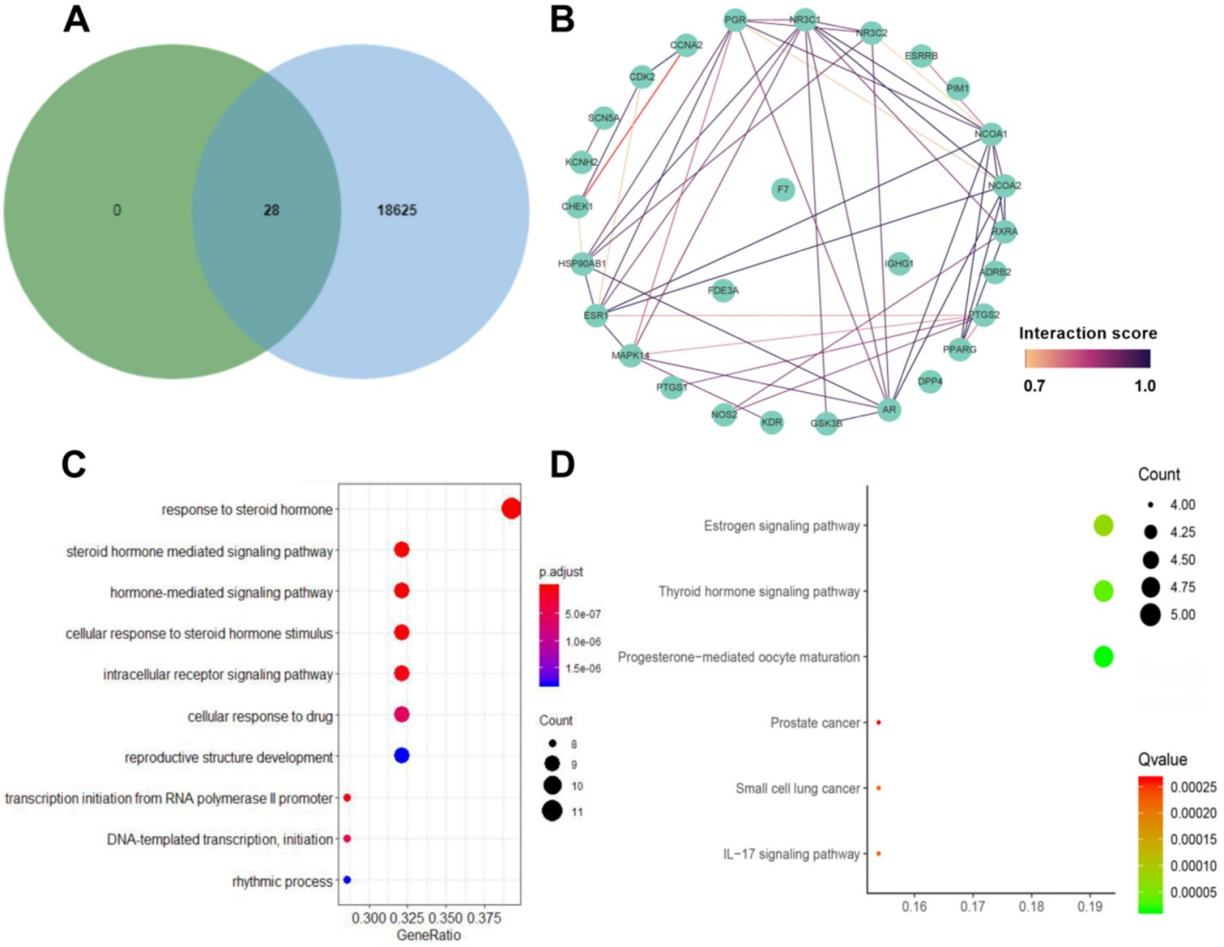
** A.** Venn diagram summaries common targets associated with diabetic nephropathy and Chuanxiong Rhizoma; **B.** Target protein interaction network of Chuanxiong Rhizoma from STRING; **C.** Gene ontology enrichment analysis of 28 specialized targets; **D.** KEGG pathway enrichment analysis of 28 specialized targets.

We searched “diabetic nephropathy” in the CTD database and found 18652 DN-related genes. Ultimately, Venn diagram summarized 28 common targets both associated with DN and Chuanxiong Rhizoma (**Fig. 3A**) for further analysis. Target protein interaction network of Chuanxiong Rhizoma from STRING was shown in **Figure 3B.**

### GO biological process analyses and KEGG pathway enrichment analyses

Twenty-eight targets fished by 6 active ingredients of Chuanxiong Rhizoma and DN from CTD database were further analyzed for functional prediction. The top 10 significant GO analyses and 6 KEGG pathways were enriched for genomic analysis. In GO analysis (**Fig. 3C**), target genes were mainly enriched in response to steroid hormone (GO: 0048545); rhythmic process (GO: 0048511); steroid hormone mediated signaling pathway (GO: 0043401); cellular response to drug (GO: 0035690); hormone-mediated signaling pathway (GO: 0009755); cellular response to steroid hormone stimulus (GO: 0071383); reproductive structure development (GO: 0048608); intracellular receptor signaling pathway (GO: 0030522); transcription initiation from RNA polymerase II promoter (GO: 0006367); DNA-templated transcription, initiation (GO: 0006352). **Figure 3D** showed the enriched KEGG pathways of targets, including progesterone-mediated oocyte maturation (hsa04914), thyroid hormone signaling pathway (hsa04919), estrogen signaling pathway (hsa04915), IL-17 signaling pathway (hsa04657), small cell lung cancer (hsa05222) and prostate cancer (hsa05215).

### Hub proteins designation and molecular docking

**Figure 4:**
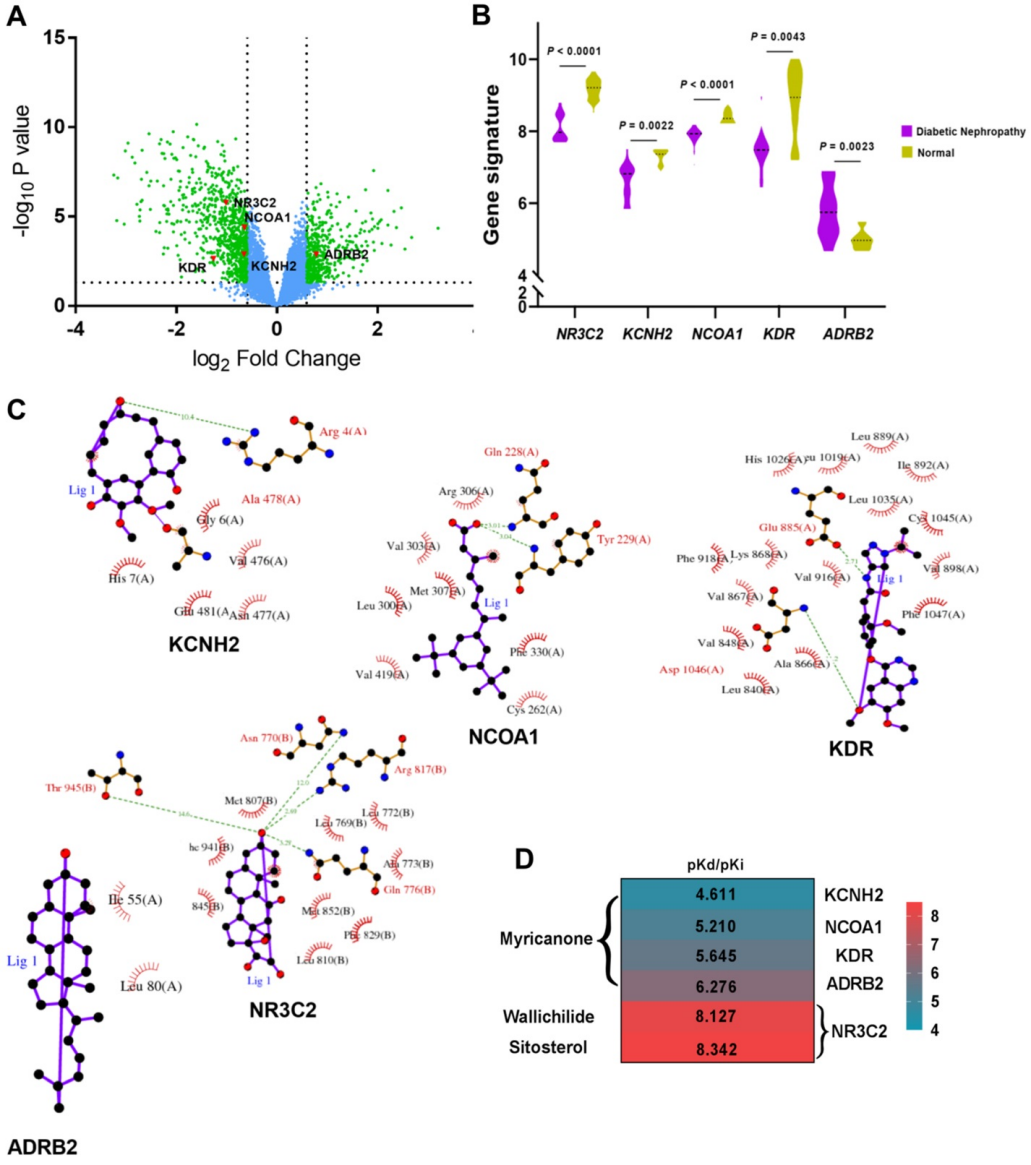
** A.** Volcano Plot represents differentially expressed genes (DEGs) associated with diabetic nephropathy from GSE 30528 dataset and 5 hub proteins (gene symbol: KCNH2, NCOA1, KDR, NR3C2 and ADRB2) targeted by Chuanxiong Rhizoma; green plots represent DEGs; red plots represent hub genes/proteins; **B.** Different gene signatures of hub genes in diabetic nephropathy patients compared with controlled patients in GSE 30528 dataset; **C.** Ligand-protein interaction diagrams of hub proteins; **D.** Heatmap shows docking scores (pKd/pKi) of hub proteins combining to active compounds of Chuanxiong Rhizoma.

Based on GSE30528 dataset, 1366 DEGs associated with DN were identified, which were shown as green plots in **Figure 4A**, including five targeted genes: KCNH2, NCOA1, KDR, NR3C2 and ADRB2 (red plots in **Fig. 4A**). Further analysis revealed that gene signatures of KCNH2, NCOA1, KDR and NR3C2 were significantly lower in DN glomeruli tissues when compared to controlled normal glomeruli tissues, while gene signature of ADRB2 was significantly higher in DN glomeruli tissues (**Fig. 4B**). The corresponding five target proteins were designed as hub proteins with ligand-protein interaction diagrams (**Fig. 4C**) for further molecular docking analysis.

With the help of SystemsDock, we successfully combined KCNH2, NCOA1, KDR and ADRB2 to Myricanone with docking scores from 4.61 to 6.28. What more, NR3C2 also displayed good binding scores with Wallichilide and Sitosterol (8.13 and 8.34, respectively, **Fig. 4D**), revealing good binding forces to active compounds of Chuanxiong Rhizoma.

### Construction of compound-target protein-pathway network of Chuanxiong Rhizoma

**Figure 5:**
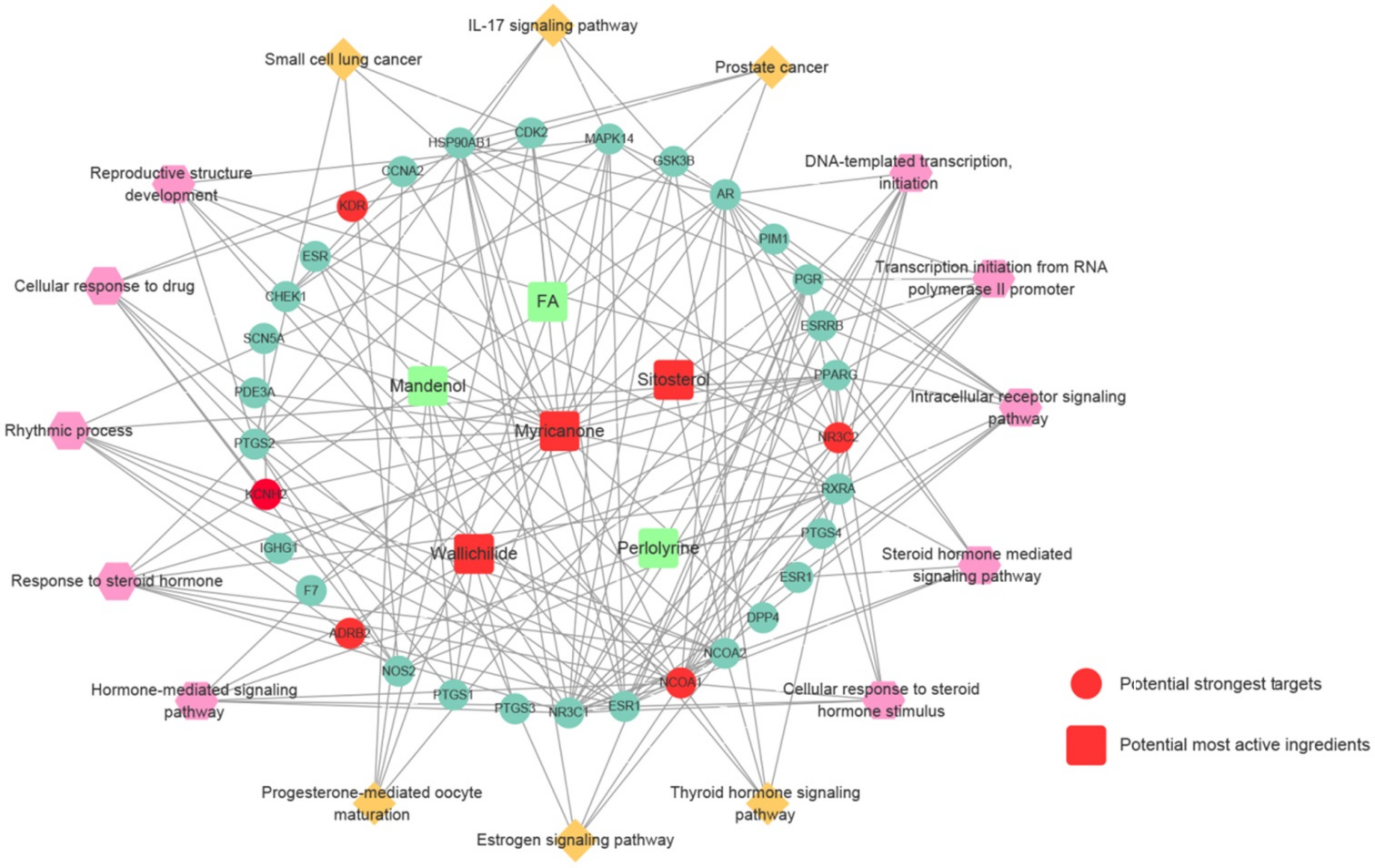
Prediction of compound-target protein-pathway network of Chuanxiong Rhizoma**.** Squires represent active compounds of Chuanxiong Rhizoma. Rotundities represent gene symbols of targets. Hexagons represent enriched gene ontology biological processes. Diamonds represent enriched KEGG pathways.

Compound-target protein-pathway network of Chuanxiong Rhizoma was constructed using Cytoscape 3.5.1 software. The interaction network was constituted by 6 active ingredients of Chuanxiong Rhizoma, 28 corresponding target proteins (shown by gene symbol), 10 GO processes and 6 KEGG pathways. As shown in **Figure 5,** squires represent active compounds of Chuanxiong Rhizoma, rotundities represent gene symbols of targets, hexagons represent enriched GO processes and diamonds represent enriched KEGG pathways.

## Discussion

As one of the major causes of ESRD around the world, DN is closely associated with cardiovascular and cerebrovascular diseases and increased mortality of DM patients 26. DN is characterized by proteinuria and changes in renal ultrastructure, and the prognosis of patients with DN is poor, especially for patients with ESRD. Due to the limitations in the understanding of DN, currently, there is still a lack of accurate treatment strategies specifically targeting at DN in addition to controlling blood sugar, blood lipid levels and hypertension.

In this study, we carried out a network pharmacology research focus on Chuanxiong Rhizoma to further explore its potential mechanisms on DN. By applying GO biological process analyses, the special targets of Chuanxiong Rhizoma were significantly enriched in hormone-mediated signaling pathway, response to steroid hormone, and other pathways related to reproductive structure development. Previous studies suggested that steroid hormone might play a vital role in the pathogenesis of DN in DM patients 27-29. Estradiol could inhibit the transcription of type IV collagen and reduce the expression of collagen through activating tyrosine kinase 2 and inhibiting the synthesis of TGF-β to alleviate fibrosis in DN patients 30. Our study revealed that Chuanxiong Rhizoma might target at steroid hormone in the treatment of DN.

KEGG pathway enrichment analyses suggested that Chuanxiong Rhizoma was associated with estrogen signaling pathway, thyroid hormone signaling pathway and IL-17 signaling pathway in the treatment of DN. Estrogen has been reported to act on renal protection by up-regulating the level of endothelial NO synthase and inhibiting the synthesis of inducible NO synthase to maintain normal renal function 31, 32. DN patients are often accompanied by hypothyroidism and low triiodothyronine syndrome. Serum free triiodothyronine acts as a key predictor of prognosis in patients with DN 33. In addition, thyroid hormone replacement therapy could reduce the risk of cardiovascular diseases in DN patients with hypothyroidism 34. Recently, the relationship between IL-17 and DN has attracted more and more attentions from the researchers. Patients with DN are reported to have elevated levels of IL-17 in their peripheral blood 35. Here, with the help of network pharmacology analysis, our study revealed that Chuanxiong Rhizoma might take part in the treatment of DN through pathways associated with estrogen, thyroid hormone and IL-17.

In our study, KCNH2, NCOA1, KDR, ADRB2 and NR3C2 are closely related to corresponding active ingredients of Chuanxiong Rhizoma with favorable molecular docking scores, indicating good binding relationships between compounds and target proteins. Among the hub targets, KCNH2 is involved in ventricular cardiac muscle cell action potential repolarization, while NCOA1 is enriched in thyroid hormone signaling pathway and estrogen signaling pathway. ADRB2 regulates beta-2 adrenergic receptor (β2AR), which has been reported to act on inhibiting of macrophage function 36 and be involved in LPS-induced activation of THP-1 cells 37. Agonists of β2AR are involved in the regulation of macrophage activation in diabetic cardiovascular and renal complications 38.

There are still some shortcomings in our study. Firstly, we just explored the potential functional mechanism of Chuanxiong Rhizoma on DN, without analyzing the mutual interferences of drug components. Secondly, this study failed to perform stratification analysis of different pathological stages of DN. Finally, the key targets and pathways we obtained have not been experimentally verified. Further molecular mechanism researches and clinical validations are still needed to be carried out in future work.

## Conclusions

Chuanxiong Rhizoma has been proven to play a role in the treatment of DN. Through network pharmacology analysis, our study revealed that Chuanxiong Rhizoma might take part in the treatment of DN through pathways associated with steroid hormone, estrogen, thyroid hormone and IL-17. KCNH2, NCOA1, KDR, ADRB2 and NR3C2 were proved to be the hub targets, which were closely related to corresponding active ingredients of Chuanxiong Rhizoma through molecular docking. However, further molecular mechanism researches and clinical validations are still needed in the future.

## Figures and Tables

**Table 1:** Characteristics of the seven active ingredients of Chuanxiong Rhizoma

Molecule ID	Molecule name	Molecular weight	OB (%)	DL
MOL000433	FA	441.45	68.96	0.71
MOL002140	Perlolyrine	264.3	65.95	0.27
MOL002151	Senkyunone	326.52	47.66	0.24
MOL002157	Wallichilide	412.57	42.31	0.71
MOL001494	Mandenol	308.56	42	0.19
MOL002135	Myricanone	356.45	40.6	0.51
MOL000359	Sitosterol	414.79	36.91	0.75

OB: oral bioavailability; DL: drug-likeness.

**Table 2:** Predicted target from active ingredients

Target Name	UniProt ID	Gene code	General Function
Nitric oxide synthase, inducible	P35228	NOS2	Tetrahydrobiopterin binding
Prostaglandin G/H synthase 1	P23219	PTGS1	Prostaglandin-endoperoxide synthase activity
Potassium voltage-gated channel subfamily H member 2	Q12809	KCNH2	Voltage-gated potassium channel activity involved in ventricular cardiac muscle cell action potential repolarization
Estrogen receptor	P03372	ESR1	Zinc ion binding
Androgen receptor	P10275	AR	Zinc ion binding
Sodium channel protein type 5 subunit alpha	Q14524	SCN5A	Voltage-gated sodium channel activity involved in sa node cell action potential
Peroxisome proliferator activated receptor gamma	P37231	PPARG	Zinc ion binding
Prostaglandin G/H synthase 2	P35354	PTGS2	Prostaglandin-endoperoxide synthase activity
Coagulation factor VII	P08709	F7	Serine-type peptidase activity
Vascular endothelial growth factor receptor 2	P35968	KDR	Vascular endothelial growth factor receptor binding
Retinoic acid receptor RXR-alpha	P19793	RXRA	Zinc ion binding
CGMP-inhibited 3',5'-cyclic phosphodiesterase A	Q14432	PDE3A	Metal ion binding
Beta-2 adrenergic receptor	P07550	ADRB2	Protein homodimerization activity
Estrogen receptor beta	O95718	ESRRB	Zinc ion binding
Dipeptidyl peptidase IV	P27487	DPP4	Virus receptor activity
Mitogen-activated protein kinase 14	Q16539	MAPK14	Protein serine/threonine kinase activity
Glycogen synthase kinase-3 beta	P49841	GSK3B	Ubiquitin protein ligase binding
Heat shock protein HSP 90	P07900	HSP90AB1	Tpr domain binding
Serine/threonine-protein kinase Chk1	O14757	CHEK1	Protein serine/threonine kinase activity
Ig gamma-1 chain C region	P01857	IGHG1	Immunoglobulin receptor binding
Proto-oncogene serine/threonine-protein kinase Pim-1	P11309	PIM1	Transcription factor binding
Cyclin-A2	P20248	CCNA2	Essential for the control of the cell cycle at the G1/S (start) and the G2/M (mitosis) transitions
Nuclear receptor coactivator 1	Q15788	NCOA1	Transcription coactivator activity
Nuclear receptor coactivator 2	Q15596	NCOA2	Transcription coactivator activity
Mineralocorticoid receptor	P08235	NR3C2	Zinc ion binding
Glucocorticoid receptor	P04150	NR3C1	Zinc ion binding
Cell division protein kinase 2	P24941	CDK2	Metal ion binding
Progesterone receptor	P06401	PGR	Zinc ion binding

## Abbreviations

DN: diabetic nephropathy
GO: Gene Ontology
DM: diabetes mellitus
ESRD: end-stage renal disease
TCM: Traditional Chinese Medicine
BUN: blood urea nitrogen
SCr: serum creatinine
TCMSP: Traditional Chinese Medicine Systems Pharmacology Database and Analysis Platform
OB: oral bioavailability
DL: drug-likeness
CTD: comparative toxicogenomics database
KEGG: Kyoto Encyclopedia of Genes and Genomes
DEGs: differentially expressed genes
PDB: Protein Data Bank

## References

[1] Sung JK, Koh JH, Lee MY, Kim BH, Nam SM, Kim JH (2010). Aldose reductase inhibitor ameliorates renal vascular endothelial growth factor expression in streptozotocin-induced diabetic rats. Yonsei Med J.

[2] Afkarian M, Zelnick LR, Hall YN, Heagerty PJ, Tuttle K, Weiss NS (2016). Clinical Manifestations of Kidney Disease Among US Adults With Diabetes, 1988-2014. Jama.

[3] Rosolowsky ET, Skupien J, Smiles AM, Niewczas M, Roshan B, Stanton R (2011). Risk for ESRD in type 1 diabetes remains high despite renoprotection. J Am Soc Nephrol.

[4] Sun GD, Li CY, Cui WP, Guo QY, Dong CQ, Zou HB (2016). Review of Herbal Traditional Chinese Medicine for the Treatment of Diabetic Nephropathy. J Diabetes Res.

[5] Ho JW, Jie M (2007). Pharmacological activity of cardiovascular agents from herbal medicine. Cardiovasc Hematol Agents Med Chem.

[6] Zhao Y, Liu Y, Chen K (2016). Mechanisms and Clinical Application of Tetramethylpyrazine (an Interesting Natural Compound Isolated from Ligusticum Wallichii): Current Status and Perspective. Oxid Med Cell Longev.

[7] Wang GF, Shi CG, Sun MZ, Wang L, Wu SX, Wang HF (2013). Tetramethylpyrazine attenuates atherosclerosis development and protects endothelial cells from ox-LDL. Cardiovasc Drugs Ther.

[8] Liang MJ, He LC, Yang GD (2005). Screening, analysis and in vitro vasodilatation of effective components from Ligusticum Chuanxiong. Life Sci.

[9] Zengyong Q, Jiangwei M, Huajin L (2011). Effect of Ligusticum wallichii aqueous extract on oxidative injury and immunity activity in myocardial ischemic reperfusion rats. Int J Mol Sci.

[10] Yang WJ, Li YR, Gao H, Wu XY, Wang XL, Wang XN (2018). Protective effect of the ethanol extract from Ligusticum chuanxiong rhizome against streptozotocin-induced diabetic nephropathy in mice. J Ethnopharmacol.

[11] Wang B, Ni Q, Wang X, Lin L (2012). Meta-analysis of the clinical effect of ligustrazine on diabetic nephropathy. Am J Chin Med.

[12] Zhang R, Zhu X, Bai H, Ning K (2019). Network Pharmacology Databases for Traditional Chinese Medicine: Review and Assessment. Front Pharmacol.

[13] Wang Y, Jafari M, Tang Y, Tang J (2019). Predicting Meridian in Chinese traditional medicine using machine learning approaches. PLoS Comput Biol.

[14] Tanoli Z, Seemab U, Scherer A, Wennerberg K, Tang J, Vähä-Koskela M (2020). Exploration of databases and methods supporting drug repurposing: a comprehensive survey. Brief Bioinform.

[15] Li Y, Zhang J, Zhang L, Chen X, Pan Y, Chen SS (2015). Systems pharmacology to decipher the combinational anti-migraine effects of Tianshu formula. J Ethnopharmacol.

[16] Wishart DS, Feunang YD, Guo AC, Lo EJ, Marcu A, Grant JR (2018). DrugBank 5.0: a major update to the DrugBank database for 2018. Nucleic Acids Res.

[17] UniProt Consortium T (2018). UniProt: the universal protein knowledgebase. Nucleic Acids Res.

[18] Berman HM, Westbrook J, Feng Z, Gilliland G, Bhat TN, Weissig H (2000). The Protein Data Bank. Nucleic Acids Res.

[19] Merico D, Isserlin R, Stueker O, Emili A, Bader GD (2010). Enrichment map: a network-based method for gene-set enrichment visualization and interpretation. PLoS One.

[20] Yu G, Wang LG, Han Y, He QY (2012). clusterProfiler: an R package for comparing biological themes among gene clusters. Omics.

[21] Woroniecka KI, Park AS, Mohtat D, Thomas DB, Pullman JM, Susztak K (2011). Transcriptome analysis of human diabetic kidney disease. Diabetes.

[22] Irizarry RA, Bolstad BM, Collin F, Cope LM, Hobbs B, Speed TP (2003). Summaries of Affymetrix GeneChip probe level data. Nucleic Acids Res.

[23] Ritchie ME, Phipson B, Wu D, Hu Y, Law CW, Shi W (2015). limma powers differential expression analyses for RNA-sequencing and microarray studies. Nucleic Acids Res.

[24] Hsin KY, Matsuoka Y, Asai Y, Kamiyoshi K, Watanabe T, Kawaoka Y (2016). systemsDock: a web server for network pharmacology-based prediction and analysis. Nucleic Acids Res.

[25] Hsin KY, Ghosh S, Kitano H (2013). Combining machine learning systems and multiple docking simulation packages to improve docking prediction reliability for network pharmacology. PLoS One.

[26] Duran-Salgado MB, Rubio-Guerra AF (2014). Diabetic nephropathy and inflammation. World J Diabetes.

[27] Maric C, Forsblom C, Thorn L, Wadén J, Groop PH (2010). Association between testosterone, estradiol and sex hormone binding globulin levels in men with type 1 diabetes with nephropathy. Steroids.

[28] Fukui M, Soh J, Tanaka M, Kitagawa Y, Hasegawa G, Yoshikawa T (2007). Low serum testosterone concentration in middle-aged men with type 2 diabetes. Endocr J.

[29] Grossmann M, Thomas MC, Panagiotopoulos S, Sharpe K, Macisaac RJ, Clarke S (2008). Low testosterone levels are common and associated with insulin resistance in men with diabetes. J Clin Endocrinol Metab.

[30] Zdunek M, Silbiger S, Lei J, Neugarten J (2001). Protein kinase CK2 mediates TGF-beta1-stimulated type IV collagen gene transcription and its reversal by estradiol. Kidney Int.

[31] Xie P, Liu ML, Gu YP, Lu J, Xu X, Zeng WM (2003). Oestrogen improves glucose metabolism and insulin signal transduction in HepG2 cells. Clin Exp Pharmacol Physiol.

[32] Baiardi G, Macova M, Armando I, Ando H, Tyurmin D, Saavedra JM (2005). Estrogen upregulates renal angiotensin II AT1 and AT2 receptors in the rat. Regul Pept.

[33] Lin Y, Sun Z (2011). Thyroid hormone ameliorates diabetic nephropathy in a mouse model of type II diabetes. J Endocrinol.

[34] Seo C, Kim S, Lee M, Cha MU, Kim H, Park S (2018). THYROID HORMONE REPLACEMENT REDUCES THE RISK OF CARDIOVASCULAR DISEASES IN DIABETIC NEPHROPATHY PATIENTS WITH SUBCLINICAL HYPOTHYROIDISM. Endocr Pract.

[35] Zhang C, Xiao C, Wang P, Xu W, Zhang A, Li Q (2014). The alteration of Th1/Th2/Th17/Treg paradigm in patients with type 2 diabetes mellitus: Relationship with diabetic nephropathy. Hum Immunol.

[36] Abrass CK, O'Connor SW, Scarpace PJ, Abrass IB (1985). Characterization of the beta-adrenergic receptor of the rat peritoneal macrophage. J Immunol.

[37] Wang W, Zhang Y, Xu M, Zhang YY, He B (2015). Fenoterol inhibits LPS-induced AMPK activation and inflammatory cytokine production through β-arrestin-2 in THP-1 cell line. Biochem Biophys Res Commun.

[38] Noh H, Yu MR, Kim HJ, Lee JH, Park BW, Wu IH (2017). Beta 2-adrenergic receptor agonists are novel regulators of macrophage activation in diabetic renal and cardiovascular complications. Kidney Int.

